# Synthesis of Biocompatible Composite Material Based on Cryogels of Polyvinyl Alcohol and Calcium Phosphates

**DOI:** 10.3390/polym14163420

**Published:** 2022-08-21

**Authors:** Rustam Sadykov, Daria Lytkina, Ksenia Stepanova, Irina Kurzina

**Affiliations:** Faculty of Chemistry, Tomsk State University, 634050 Tomsk, Russia

**Keywords:** calcium phosphates, hydroxyapatite, cryogel, polyvinyl alcohol, biocompatible material

## Abstract

At the moment, the field of biomedical materials science is actively developing, which aims at creating new functional materials. A developing direction in biomedical materials science is that towards the treatment of diseases associated with bone tissue disorders, using biodegradable composite materials based on polymer and calcium phosphate materials. We developed a material based on polyvinyl alcohol cryogel, mineralized with calcium phosphate. A material based on cryogel of polyvinyl alcohol mineralized with calcium phosphate was developed. The composites were obtained by the method of cyclic freezing–thawing, and the synthesis of calcium phosphates was carried out in situ with heating, stirring, and exposure to microwave radiation. The phase composition, as well as the composition of functional groups, was determined by IR spectroscopy and X-ray phase analysis. Monocytes isolated from human blood showed higher viability compared to the controls.

## 1. Introduction

Bone defects caused by various infectious diseases, trauma, and genetic factors can lead to dysfunction and problems with the musculoskeletal system. Traditional methods of treatment, such as autologous bone grafting, face such problems as high trauma and the risk of infection, which limit their clinical use [1]. However, bone tissue engineering, namely the use of scaffolds for seeding cells and bioactive factors that allow the restoration and regeneration of bone tissue, avoids these disadvantages [2]. Scaffolds used in bone engineering should have such properties as biocompatibility, bioactivity, high porosity, mechanical strength, and biomimeticity [3,4]. Based on this, we conclude that single-component scaffolds cannot fully meet the requirements for the restoration of bone defects [5].

Bone tissue is a solid and dense substance, consisting mainly of two parts: cortical bone and spongy bone [6]. According to the chemical composition, the bone comprises an organic part, which can be attributed to collagen, and an inorganic part, which includes hydroxyapatite [7]. The creation of materials for implants that combine organic and inorganic components that can provide bone tissue regeneration is one of the main problems in biomedicine. Such materials should promote cell growth, as well as have a structural and mechanical properties similar to bone tissue [8,9].

Hydroxyapatite with the general formula Ca_10_(PO_4_)_6_(OH)_2_ is the main inorganic component of bone tissue [7]. This material has high biocompatibility, bioactivity, is non-toxic [10], and is also capable of improving the mechanical properties of scaffolds [11]. The biological activity of hydroxyapatite under physiological conditions is due to the formation of a calcium phosphate layer on its surface; in such layers, calcium phosphates differ in their structure and composition due to the fact that, during precipitation, they interact with ions in the body. In addition to improving cell proliferation and adhesion [12,13], it can also promote osteogenic differentiation of mesenchymal stem cells [14].

However, despite all the advantages of hydroxyapatite, its use in bone implants is limited [15]. This is due to the low rate of bone resorption under physiological conditions, since the processes of osteogenesis and resorption are connected; when one of the processes slows down, the other one also slows down [16]. The second reason limiting the use of hydroxyapatite is its brittleness [17]; however, it retains a very high compressive strength (up to 917 MPa) [18] and has a very weak tensile property. The reason for the fragility of this material is the ionic bond between the atoms in the ceramic material: they are not capable of plastic deformation, and the fracture toughness of ceramic composites does not exceed 1.2 MPa while the strength of bones ranges from 2 to 12 Mpa [18]. For this reason, research is currently relevant, according to the results of which it will be possible to create composite materials for biomedical purposes, which will combine biological activity and mechanical properties comparable to bone tissue [19]. Composite materials of hydroxyapatite with synthetic biocompatible polymers can improve the mechanical and biocompatible properties of hydroxyapatite [20,21].

Polyvinyl alcohol (PVA) is a synthetic biocompatible and non-toxic polymer whose cryogels have a porous structure [22,23]. The main problem when using this material for bone implantation is the fixation of the material in the body, due to its biological inertness [24]. This problem can be corrected by adding bioactive and biocompatible components to the hydrogel, which include hydroxyapatite. The combination of the macroporous and elastic structure of polyvinyl alcohol cryogel, and the biologically active and biologically compatible calcium phosphate, will lead to a synergistic effect of the functional properties of biocompatible composites [21,25].

Cryogels have important advantages in bone tissue repair. Due to the fact that cryogels consist of three-dimensional hydrophilic polymer chains, which have high mechanical strength, they are able to create a nutrient medium for the growth of endogenous cells. They are also able to imitate the extracellular matrix of bone, which makes it possible to encapsulate bioactive cells and molecules. Due to their network structure, cryogels can control the release of materials as needed [26]. In addition, cryogels are able to dissolve and show high integration with body tissues, which, as a result, makes it possible to avoid difficulties during surgical removal [27]. The mechanical properties of cryogels can be adjusted, depending on the application, by changing the polymer concentration, cooling rate, and freezing time [28]. Being a porous material, the pore size of a cryogel can vary, which makes it possible to include fillers in them, including hydroxyapatite powders [29]. Based on this, the creation of new composite materials is currently becoming an important and promising approach to bone tissue regeneration. The purpose of this work is to obtain cryogel composite materials based on polyvinyl alcohol and calcium phosphates, as well as to study the physicochemical, mechanical, and functional properties.

## 2. Materials and Methods

To obtain materials K1 and K2, a suspension of calcium hydroxide in water (3.68 g in 20 mL) was added to a 30% polyvinyl alcohol gel in water (15 mL) and stirred for 2 h. After two hours of stirring, to obtain material K1, a solution of ammonium hydrogen phosphate (3.95 g in 10 mL) was added and stirred for 6 h, and to obtain K2, an 87% solution of phosphoric acid was added and stirred for 4 h. Further, the resulting suspensions were treated with microwave radiation (power 100 W), kept for two days to preserve the precipitate, and frozen for 12 h at −20 °C. Using the same methods, but without PVA, pure HA(K1) and HA(K2) hydroxyapatite was synthesized to compare the viability of monocytes in the presence of materials (Table 1).

The phase composition of the resulting composite materials based on cryogels of polyvinyl alcohol and calcium phosphates was determined on a Rigaki diffractometer (MiniFlex 600) at 40 kV and speed 3°/min. Phase interpretation and identification was carried out using the ICDD diffraction database (PDF-2/Release 2012 RDB). The spectra of the surface layer of samples of various materials were recorded on a Nicolet 6700 IR Fourier spectrometer (Thermo Scientific). The spectra were recorded with a resolution of 4 cm^−1^ in the range 400–4000 cm^−1^ using an ATR attachment.

The surface morphology of composite materials based on cryogels of polyvinyl alcohol and calcium phosphates was studied using a HITACHI TM-3000 electron microscope at an accelerating voltage of 5 kV, without applying metals to the surface. The particle size was calculated by the linear-intercept method, in which the size measurement is made using a straight line at one selected angle for all particles, followed by plotting a diagram from the resulting data array.

To assess the solubility of HA in the composition of the composites, the total concentration of Ca^2+^ calcium ions in physiological saline at 37 °C, in which the samples were kept for 20 days to achieve saturation with respect to the solid phase, was determined by complexometric titration in the presence of Eriochrome Black T with an ammonia buffer solution, pH = 10. Titration was carried out every 7 days.

The compressive strength was measured, and the modulus of elasticity was calculated on a setup assembled on the basis of an Arduino Board Mega 2560 and a strain gauge with a sample deformation of 20%.

To determine the assessment of the effect of materials on the viability of cells of the immune system, a cytotoxicity test with an Alamar Blue indicator was used. The signal intensity was measured using a Tecan Infinite 200 microreader at a wavelength of 540 nm.

## 3. Results and Discussion

**Figure 1:**
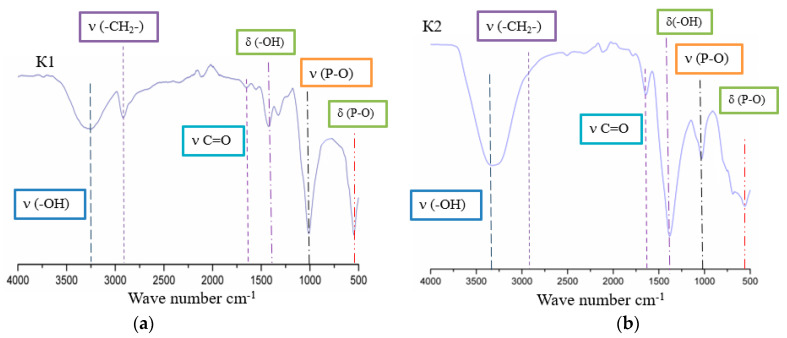
IR spectra of samples of composites K1 (**a**) and K2 (**b**).

Analysis of the resulting composites by IR spectroscopy showed that the spectra (Figure 1) have band characteristics of both hydroxyapatite and PVA. The IR spectra of the composites contain bands corresponding to bond vibrations in the structure of PVA and HA. In the composite material, there is an absorption band in the region of 3300 cm^−1^, which indicates the stretching vibrations of the O-H bond, which is a characteristic band for alcohols. The absorption at 2900 cm^−1^ refers to the stretching vibrations of the CH_2_ bond. Absorption is observed in the region of 1600 cm^−1^, which is characteristic of the stretching vibrations of the C=O bond: this band could appear namely as a result of the production of polyvinyl alcohol, during the saponification of polyvinyl acetate. An absorption band is also observed in the region of 1385 cm^−1^, which is also characteristic of the bending vibrations of O-H groups, but for the K2 sample, an increase in the intensity of this band can be observed. This is due to the formation of a larger number of hydrogen bonds. In the spectrum of hydroxyapatite, an intense absorption band is observed in the region of 1000 cm^−1^, which refers to the stretching vibrations of phosphate P-O groups, and the bands at 570 cm^−1^ refer to their deformation vibrations.

**Figure 2:**
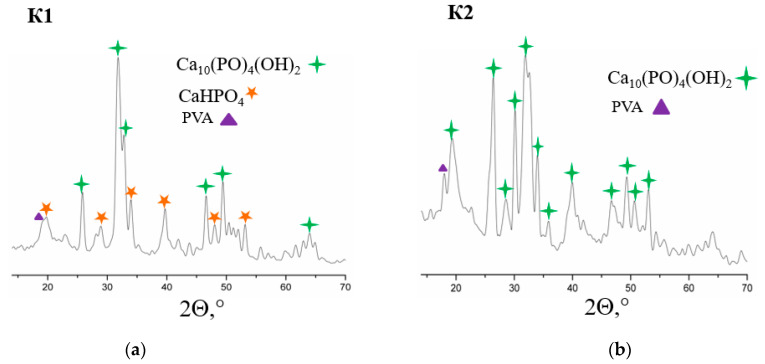
X-ray diffraction patterns of samples of composites K1 (**a**) and K2 (**b**).

When examining samples by X-ray phase analysis (Figure 2), we can observe the presence of several phases in composites. The resulting composites are crystalline. Composite K1 is a phase of hydroxyapatite and monetite. Monetite is an acidic salt of phosphoric acid. Under physiological conditions, these types of phosphates are hydrolyzed with the formation of macroporous carbon-substituted hydroxyapatite, which is characterized by a slower resorption rate compared to hydrophosphates, and acts as a fixing agent. Composite K2 is represented only by the hydroxyapatite phase, which indicates the completion of the reaction. The average crystallite size is 38–40 nm.

**Figure 3:**
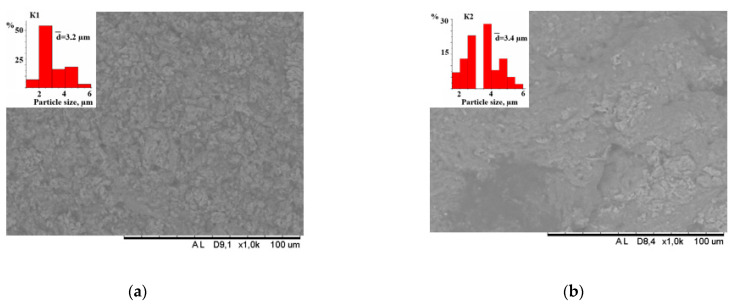
SEM image and histograms of composites K1 (**a**) and K2 (**b**).

The results of surface morphology studies by SEM showed that the K1 composite is more homogeneous compared to the K2 sample. On the histograms, we can observe that the average particle size differs slightly and is 3 µm (Figure 3). Particle size analysis was performed on the images obtained on the surface and on the cut of the sample; no significant differences in size between different particle locations were found.

Composites K1 and K2, obtained by mineralization of the polymer matrix in situ, were divided into several parts and immersed in saline, after which they were placed in a thermostat at a temperature of 37 °C and kept for four weeks. In total, four series of tests were made, and after each week, the physiological solution in which the composites were kept was titrated for the calcium content in the solution. The physiological solution was titrated with a solution of 0.01 M Trilon-B, with the indicator Eriochrome Black. From the results of the titration, we can observe that the release of calcium in the K1 composite is higher in all test series, relative to the K2 composite. For composite K1, complete diffusion occurs as early as the third week, and does not change further. The reason for the low release of calcium in the K2 composite may be the presence of only hydroxyapatite, which is a poorly soluble compound and entails low diffusion (Table 2).

**Figure 4:**
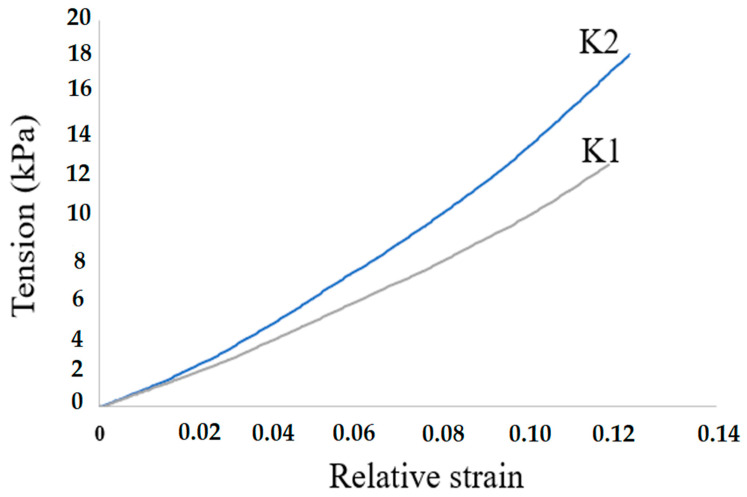
Dependences of the relative deformation of samples of composites K1 and K2 on stress.

The mechanical compressive resilience of composite materials was determined by measuring the relative stress and strain of the composites. Based on the measurement results, dependency graphs were obtained (Figure 4).

0.02 Based on the results of these measurements, the graphs were processed, and the average modulus of elasticity was calculated (Table 3). Based on the results of these studies, we can observe that the average modulus of elasticity of the K2 composite is higher in comparison with the K1 composite. This is due to the fact that pure hydroxyapatite is formed in the K2 composite, which, due to its hydroxyl groups, forms a greater number of crosslinking points, which leads to an increase in the elasticity of the composite.

**Figure 5:**
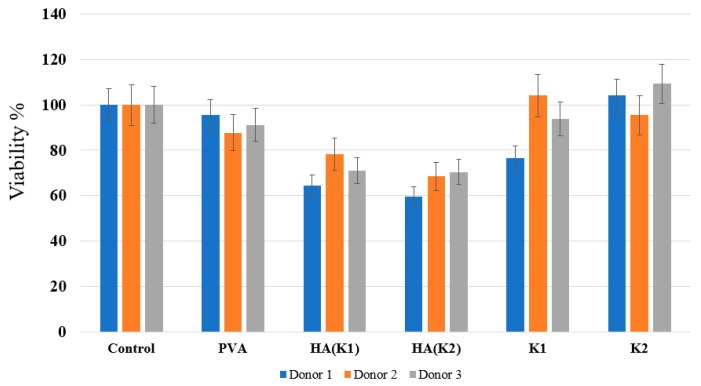
Study of the viability of macrophages in the presence of composite materials K1 and K2, pure hydroxyapatite HA (K1) and HA (K2), and pure PVA on three donors.

When analyzing the viability of monocytes in the presence of materials, in addition to composite materials, pure hydroxyapatite was also synthesized according to two methods (Figure 5). First, monocytes isolated from human blood were seeded on the samples. Monocytes were isolated from the blood of three donors. Next, the samples were incubated at 37 °C for 6 days. After that, the Alamar Blue indicator was added, and the samples were incubated for another three hours. At the final stage, fluorescence was measured. The study of the viability of macrophages in the presence of materials on three donors showed that, in the presence of pure HA, regardless of the method of preparation, about 60–80% of cells survive, while in the presence of pure PVA and composites, the level of viability is comparable to the control sample (cells without material) and some donors are higher than the control. This result shows that the material does not interfere with cell survival, which indicates a low cytotoxicity of the materials. The fact that, in some samples, the viability of monocytes is higher than in the control indicates that fewer cells survived in the control, and there is a more comfortable environment in the presence of the material than without it.

## 4. Conclusions

Composite materials based on a 10% solution of polyvinyl alcohol mineralized with hydroxyapatite, synthesized from a solution of calcium hydroxide, ammonium hydrogen phosphate, and phosphoric acid were obtained. It was established by X-ray phase analysis that the main phase of the mineral filler of the composite is hydroxyapatite Ca_10_(PO_4_)_6_(OH)_2_; however, in K1 there is also a phase of calcium hydroxide Ca(OH)_2_, and in K2 there is a phase of calcium hydrophosphate CaHPO_4_. The release of calcium in composite K2 occurs much faster due to the more soluble phase of calcium hydrogen phosphate CaHPO_4_. For composite K2, the average modulus of elasticity is 157 kPa, which is significantly higher than that of K1, whose average modulus of elasticity is 101 kPa. The study of the surface morphology of HA/PVA showed the presence of a homogeneous structure of the K1 composite and an inhomogeneous structure of the K2 composite; the average particle size is 3 µm. The study of the viability of monocytes showed that about 60–80% of cells survive in the presence of pure HA, and in the presence of pure PVA and composites, it is comparable with the control sample. As we can see, the resulting materials do not have outstanding mechanical properties, but can be considered biocompatible since the cells of the immune response monocytes show high viability in the presence of materials. Such a material can be used to fill the voids that have formed due to pathological processes in the bone, between the bone, and the implant bearing a mechanical load.

## Figures and Tables

**Table 1:** Composition of composite materials K1 and K2.

Name	Reaction Equation	PVA Content, %	HA Content, %
K1	10Ca(OH)_2_ + 6(NH_42_HPO_4_ → Ca_10_(PO_4_)_6_(OH)_2_↓ + 6H_2_O + 12NH_4_OH	10	10
K2	10Ca(OH)_2_ + 6H_3_PO_4_ = Ca_10_(PO_4_)_6_(OH)_2_↓ + 6H_2_O + 12NH_4_OH	10	10

**Table 2:** Solubility of composite materials K1 and K2.

	Concentration Ca^2+^ (mmol/L)			
	1 Week	2 Weeks	3 Weeks	4 Weeks
K1	4.5	4.1	5.7	5.7
K2	1.9	1.3	1.2	3.5

**Table 3:** Modulus of elasticity of composite materials K1 and K2.

	Elastic Modulus (kPa)
K1	101 ± 2
K2	157 ± 1
